# Testosterone Delivered with a Scaffold Is as Effective as Bone Morphologic Protein-2 in Promoting the Repair of Critical-Size Segmental Defect of Femoral Bone in Mice

**DOI:** 10.1371/journal.pone.0070234

**Published:** 2013-08-05

**Authors:** Bi-Hua Cheng, Tien-Min G. Chu, Chawnshang Chang, Hong-Yo Kang, Ko-En Huang

**Affiliations:** 1 Department of Obstetrics and Gynecology, Chiayi Chang Gung Memorial Hospital, Chiayi, Taiwan; 2 Department of Obstetrics and Gynecology, Kaohsiung Chang Gung Memorial Hospital, Chang Gung University College of Medicine, Kaohsiung, Taiwan; 3 Graduate Institute of Clinical Medical Sciences, College of Medicine, Chang Gung University, Tao-Yuan, Taiwan; 4 Center for Menopause and Reproductive Medicine Research, Kaohsiung Chang Gung Memorial Hospital, Chang Gung University College of Medicine, Kaohsiung, Taiwan; 5 Department of Restorative Dentistry, Indiana University School of Dentistry, Indianapolis, Indiana, United States of America; 6 George Whipple Lab for Cancer Research, Departments of Pathology, Urology, Radiation Oncology, and The Wilmot Cancer Center, University of Rochester Medical Center, Rochester, New York, United States of America; Georgia Health Sciences University, United States of America

## Abstract

Loss of large bone segments due to fracture resulting from trauma or tumor removal is a common clinical problem. The goal of this study was to evaluate the use of scaffolds containing testosterone, bone morphogenetic protein-2 (BMP-2), or a combination of both for treatment of critical-size segmental bone defects in mice. A 2.5-mm wide osteotomy was created on the left femur of wildtype and androgen receptor knockout (ARKO) mice. Testosterone, BMP-2, or both were delivered locally using a scaffold that bridged the fracture. Results of X-ray imaging showed that in both wildtype and ARKO mice, BMP-2 treatment induced callus formation within 14 days after initiation of the treatment. Testosterone treatment also induced callus formation within 14 days in wildtype but not in ARKO mice. Micro-computed tomography and histological examinations revealed that testosterone treatment caused similar degrees of callus formation as BMP-2 treatment in wildtype mice, but had no such effect in ARKO mice, suggesting that the androgen receptor is required for testosterone to initiate fracture healing. These results demonstrate that testosterone is as effective as BMP-2 in promoting the healing of critical-size segmental defects and that combination therapy with testosterone and BMP-2 is superior to single therapy. Results of this study may provide a foundation to develop a cost effective and efficient therapeutic modality for treatment of bone fractures with segmental defects.

## Introduction

Bone fracture is a common and serious medical problem. Fractures may occur in any bone due to high force impact and are much more prevalent in individuals with osteoporosis, bone cancer, or osteogenesis imperfecta. The more severe type of fracture is the loss of a segment or portion of bone, commonly referred to as segmental defect which may be caused by trauma, tumor removal, or surgery for congenital deformation. Segmental defects are very difficult to manage, as multiple phases of surgery are usually required to achieve an adequate union and to regain the function of bone. Although current treatment options such as autografts, allografts, and distraction osteogenesis have yielded some successes [1]–[3], serious consequences such as leg shortening or amputation may result if the treatment fails. To overcome these limitations, tissue engineering by delivering therapeutic substances such as bone morphogenetic proteins (BMPs) to the segmental defect to facilitate bone regeneration has been attempted [4]–[6].

BMPs are members of the TGF-β superfamily [7]. Among the several BMPs, BMP-2 has been shown to induce differentiation of mesenchymal cells into chondroblasts and osteoblasts [8], [9], endochondral ossification and chondrogenesis, expression of type II and X collagens, and incorporation of sulfate into glycosaminoglycans in growth plate cell cultures [10]. BMP-2 was originally shown to promote the healing of critical-size fracture defects in a goat tibia fracture model and a rabbit ulnar osteotomy model [4], [5]. In these models, BMP-2 was delivered to the fracture by wrapping the traumatized bone with an absorbable collagen sponge containing recombinant human BMP-2 (rhBMP-2). Other materials, such as hydrogels and calcium phosphate cements, have also been used to deliver BMP-2 to enhance fracture healing [11], [12].

The rhBMP-2, under the trade name INFUSE, has been approved by the Food and Drug Administration for use with the lumbar tapered fusion device to perform single-level anterior lumbar inter body fusions (ALIF) [13] and treatment of acute, open tibial shaft fractures [14]. However, rhBMP-2 is also widely used off-label for anterior and posterior cervical, thoracic, and lumbar surgery. Although its use in ALIF is generally satisfactory [15], a number of complications such as ectopic bone formation [16], neurological deterioration [17], respiratory failure [18], and inflammation of adjacent tissues [17] have been associated with rhBMP-2 in off-label applications. Furthermore, clinical applications of BMP-2 for fracture repair are often prohibitive due to its high cost [13], [19]. Therefore, development of alternative means to aid fracture repair is warranted. In this study, we tested whether testosterone can replace BMP-2 for treatment of segmental fractures.

Testosterone is one form of androgens. They mediate their effects mainly through binding to the androgen receptor (AR) [20] that functions as a ligand-inducible transcription factor controlling an integrated gene expression program required for bone formation and mineralization [21]. Previous reports showed that deficiency of androgen receptor in male mice results in osteopenia, bone loss, and adverse changes in calvaria, tibia, and femur [22]–[24]. Androgens are considered bone anabolic agents as androgen deficiency or hypogonadism in men is associated with increased bone turnover and bone loss, and these defects can be reversed after treatment with androgens [25]. While systemic treatment with androgens have been shown to increase bone mass and promote the healing of bone defects in animal models [26], [27], the effect of local short-term testosterone treatment delivered with bio-degradable scaffolds on the healing of segmental bone defects remains unclear.

We have previously developed a load-bearing biodegradable scaffold made of polypropylene fumarate/tricalcium phosphate composites and have shown its effectiveness in promoting the healing of critical-size segmental defects [6]. In this study, we investigated the possibility of using the scaffold containing testosterone to promote the healing of nonunion fractures. We also compared its efficacy with that of BMP-2 or combination of both testosterone and BMP-2 for fracture repair.

## Materials and Methods

### Animals

Eight weeks old of both wildtype (AR^+/Y^) and androgen receptor knockout (ARKO; AR^−/Y^) male C57BL/6 mice were used in this study. The ARKO mice [28] were originally obtained from George H. Whipple Lab for Cancer Research, University of Rochester and bred in Chang Gung Memorial Hospital animal facility. All animal procedures were done according to the Guide for the Care and Use of Laboratory Animals published by the Institute of Laboratory Animal Resources, National Research Council, National Academy of Science, Taiwan. This study was approved by the Animal Care and Use Committee of the Chang Gung Memorial Hospital, Kaohsiung, Taiwan. Animals were housed in pathogen-free facilities on a 12-h light and dark schedule with light on at 6 a.m.

### Bone scaffolds

Scaffolds were produced as described previously [6]. Briefly, a thermal-curable polypropylene fumarate (PPF)/tricalcium phosphate (TCP) suspension was prepared by mixing PPF, N-vinyl pyrrolidinone, and TCP at a weight ratio of 1∶0.75∶0.66. The PPF/TCP slurry was then mixed with 0.5% benzoyl peroxide (thermal initiator) and 10 ml of dimethyl p-toluidine (accelerator), and cast into a wax mold to produce tube-shaped structures (outer diameter, 4 mm; inner diameter, 2 mm; height 2.5 mm, with two side holes of 800 µm in diameter). These scaffolds were load-bearing as they allowed mobility of fractured bone when applied [6]. Each scaffold was loaded with rhBMP-2 (Wyeth, Cambridge, MA) by soaking it in 5 µl of 1 µg/µl rhBMP2 in phosphate buffered saline or with testosterone (Sigma-Aldrich, St. Louis, IL) by soaking it in 1 ml of absolute ethanol containing 100 µg of testosterone overnight. The scaffolds that contained both BMP-2 and testosterone were treated with testosterone first and then with rhBMP-2. The dose of rhBMP-2 (5 µg/scaffold) used was chosen based on previous studies [6], [29]. 100 µg of testosterone per scaffold was used because this dose is equivalent to 1.8 ng/ml in humans, approximately the lower limit of physiological circulating concentration in men (2–9 ng/ml) [30], [31].

### Generation of critical-size segmental defect on femoral bone

To investigate the effects of BMP-2 and testosterone on the healing of segmental defects, twenty-eight each of 8 weeks old wildtype C57BL/6 and ARKO (in C57BL/6 background) male mice were used. The mice were anesthetized by intra-peritoneal injection of xylazine (10 mg/kg) and ketamine (80 mg/kg). Buprenorphine (0.1 mg/kg) was given by intra-muscular injection on the non-operated leg as analgesic agent. A segmental defect of approximately 2.5 mm was generated on the left femur of each mouse using a rotating blade with copious irrigation. A 2.5-mm PPF/TCP scaffold was inserted to bridge the fracture. Each scaffold was loaded with PBS (Blank), BMP-2 (5 µg), testosterone (100 µg), or BMP-2 (5 µg) plus testosterone (100 µg). A 27-gauge stainless steel needle was used as an intramedullary pin. The needle was drilled into the trochlear groove between the lateral and medial condyles to reach the femur marrow cavity. It was allowed to pass through the central channel of the scaffold and attached to the proximal end of the femur marrow cavity. After thorough irrigation of the operation field, the muscles were closed in layers with 3-0 Vicryl sutures. The skin was closed with 3-0 Prolene. The mice were then returned to cages without restriction in movements.

### Radiography

After closure of the surgical wound, the femur with segmental defect of each mouse was X-rayed at days 1, 7, 14, 21, 28, and 35 to examine the status of fracture healing.

### Micro-computed tomography

At the end of study (35 days post fracture surgery), the mice were sacrificed. The left femur with scaffold of each mouse was retrieved to quantitate callus formation by micro-computed tomography (micro-CT). The right femur without scaffold was similarly examined to serve as control. Micro-CT was performed using a Skyscan 1076 instrument (Skyscan) [32]. Each femur was scanned for 260 slices each above and below the midpoint of the scaffold for a total of 8.2 mm in length. Three dimensional reconstruction and quantitative analyses of callus and mineralized bone volumes; trabecular thickness, number, and separation; structure model index; cortical bone mineral density, thickness, and degree of anisotropy were performed using NRecon, ANT, and CATn (version 1.4) software packages supplied with the Skyscan instrument.

### Histology

After retrieval, the femurs were placed in 10% buffered formalin, dehydrated with 70–100% alcohol, and then embedded in poly(methyl methacrylate). To examine fracture healing, longitudinal sections of the region with scaffold were made and stained with hematoxylin and eosin (H&E) to reveal the general structure of tissue or with Van Kossa/MacNeal staining to examine trabecular and cortical bones as described previously [33]. For Van Kossa/MacNeal staining, sections on slides were rehydrated and then transferred to a solution containing 1% silver nitrate for 15–60 minutes under a strong light until blackening of the mineralized bone was seen. Sections were then gently washed three times with distilled water followed by treatment with thiosulfate for 5 min. After washing with water, the sections were counterstained for 3 minutes with methyl green and rinsed three times with n-butanol. The sections were further counterstained with MacNeal stain by immersing the slides in freshly prepared 5% tetrachrome for 20–60 min, rinsed twice with 2-propanol, soaked for 5 minutes in xylol, and finally coverslipped with the Roti-histo kit. To detect osteoclasts, TRAP (tartrate-resistant acid phosphatase) staining was performed using the Acid Phosphatase, Leukocyte kit (Sigma-Aldrich) as acid phosphatase is a marker of osteoclasts [34]. The sections were incubated in naphthol AS-BI-phosphoric acid-acetic acid-tartrate solution (pH 5.2) for 1 h at 37°C and then counterstained with acid hematoxylin. The sections were then washed with distilled water and mounted.

### Statistical analyses

All values shown were mean with standard deviation (SD) (n = 7). Differences among groups were assessed by the Wilcoxon/Kruskal-Wallis test followed by post-Hoc test with Dunn's method. In all statistical comparisons, p<0.05 was defined as a significant difference. The SigmaStat statistics software (version 15.0; SPSS) was used for all calculations.

## Results

### Radiographic analyses on effects of BMP-2, testosterone, or combination of both BMP-2 and testosterone on the healing of segmental defects

**Figure 1:**
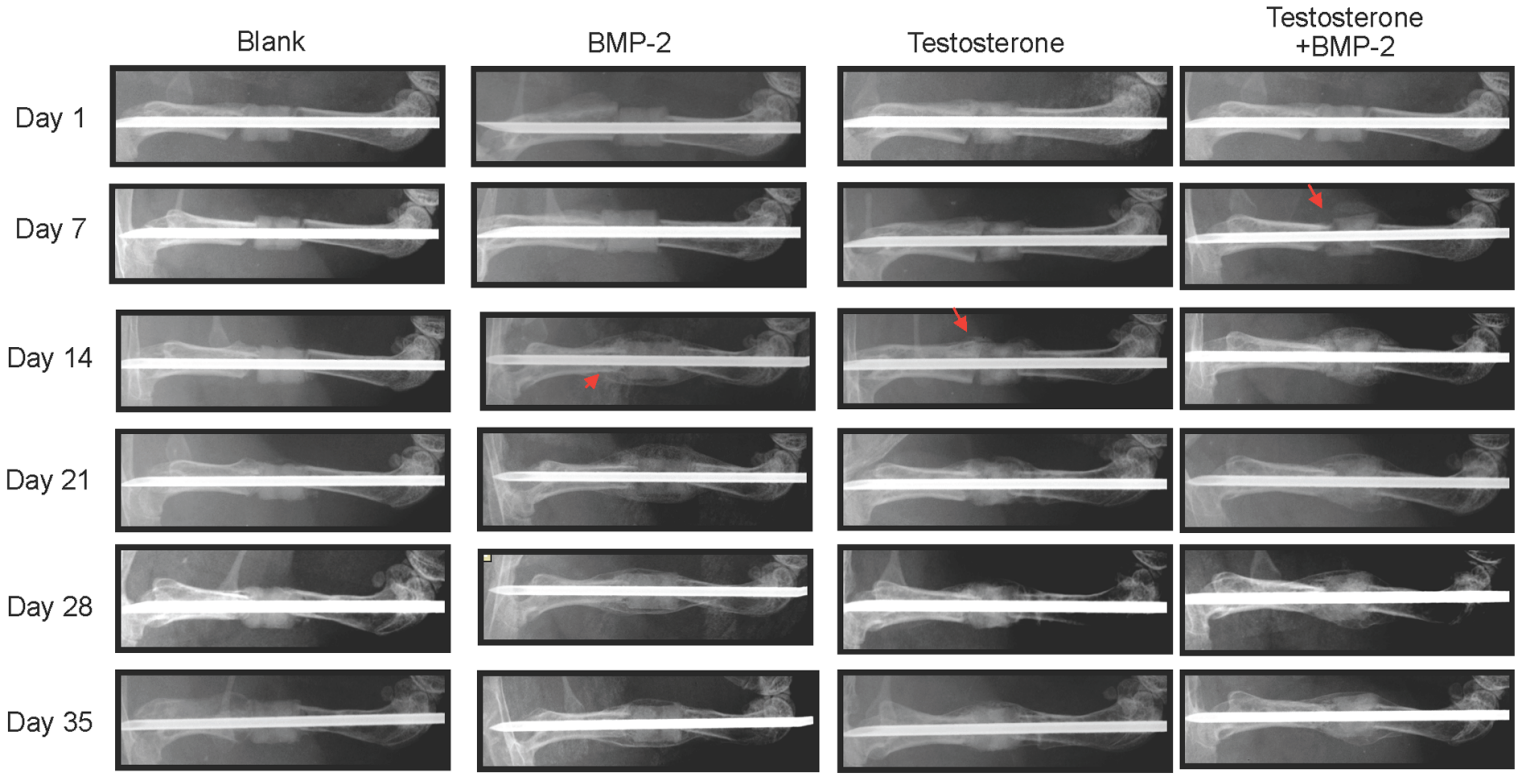
Representative serial X-ray images of left femurs of wildtype mice (AR^+/Y^) with a critical-sized segmental bone defect treated with a 2.5-mm scaffold containing BMP-2 (5 µg), testosterone (100 µg), or testosterone plus BMP-2. Arrow head: callus formation.

The femur with segmental defect of each mouse was X-rayed at days 1, 7, 14, 21, 28, and 35 after initiation of therapy to examine callus formation. As seen in Fig. 1 and Table 1, no callus formation on the fractured femur of either wildtype (7 of 7 mice) or ARKO (7 of 7 mice) mice was observed during the entire 35-day period of the study if the scaffold was loaded with PBS only. In wildtype mice, callus formation was observed on day 14 if the scaffold was loaded with BMP-2 (7 of 7 mice) or testosterone (6 of 7 mice). If the scaffold was loaded with both BMP-2 and testosterone, one mouse had callus formation 7 days earlier than others that had callus formation at day 14.

**Table 1:** Percent (and number) of wildtype and ARKO mice with callus formation on femurs with segmental defect treated with a 2.5-mm scaffold containing BMP-2 (5 µg), testosterone (100 µg), or testosterone plus BMP-2 at days 1, 7, 14, 28 and 35 after treatment.

Day	Wildtype				ARKO			
	Blank	BMP-2	T	T+B	Blank	BMP-2	T	T+B
1	0 (0/7)	0 (0/7)	0 (0/7)	0 (0/7)	0 (0/7)	0 (0/7)	0 (0/7)	0 (0/7)
7	0 (0/7)	0 (0/7)	0 (0/7)	14 (1/7)	0 (0/7)	0 (0/7)	0 (0/7)	0 (0/7)
14	0 (0/7)	100 (7/7)	86 (6/7)	100 (7/7)	0 (0/7)	100 (7/7)	0 (0/7)	100 (7/7)
21	0 (0/7)	100 (7/7)	100 (7/7)	100 (7/7)	0 (0/7)	100 (7/7)	0 (0/7)	100 (7/7)
28	0 (0/7)	100 (7/7)	100 (7/7)	100 (7/7)	0 (0/7)	100 (7/7)	0 (0/7)	100 (7/7)
35	0 (0/7)	100 (7/7)	100 (7/7)	100 (7/7)	0 (0/7)	100 (7/7)	0 (0/7)	100 (7/7)

**Figure 2:**
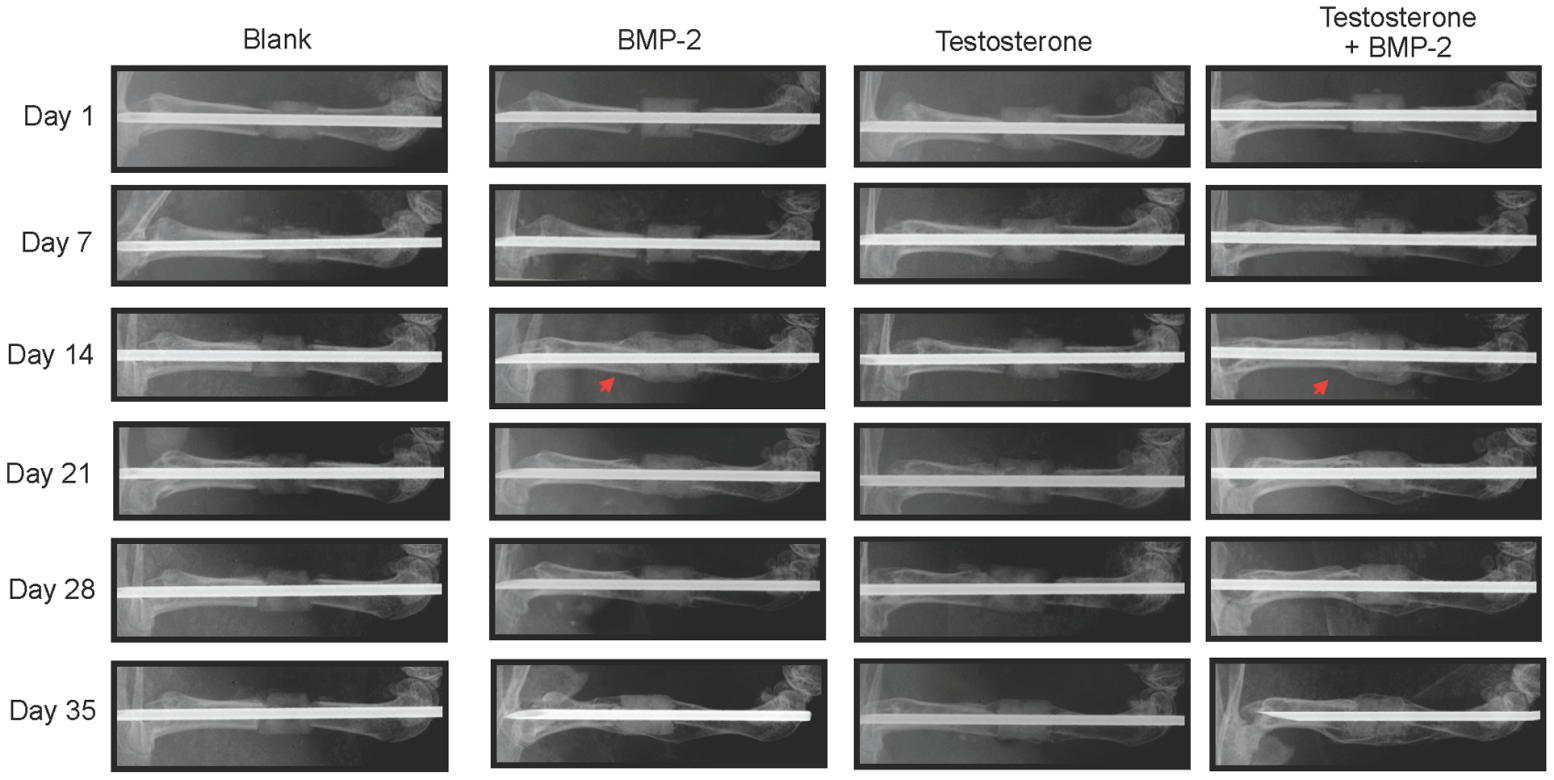
Representative serial X-ray images of left femurs of ARKO (AR^−/Y^) mice with a critical-sized segmental bone defect treated with a 2.5-mm scaffold containing BMP-2 (5 µg), testosterone (100 µg/µl), or testosterone plus BMP-2. Arrow head: callus formation.

In ARKO mice, callus was also observed on day 14 on the fractured femur treated with scaffolds containing BMP-2 (7 of 7 mice) or BMP-2 plus testosterone (7 of 7 mice) (Fig. 2 and Table 1). However, no callus was observed in all (7 of 7) ARKO mice for the entire 35-day period of the study if the fracture was treated with scaffolds containing only testosterone.

### Micro-CT analyses on effects of BMP-2, testosterone, or combination of both BMP-2 and testosterone on callus and bone formation

**Figure 3:**
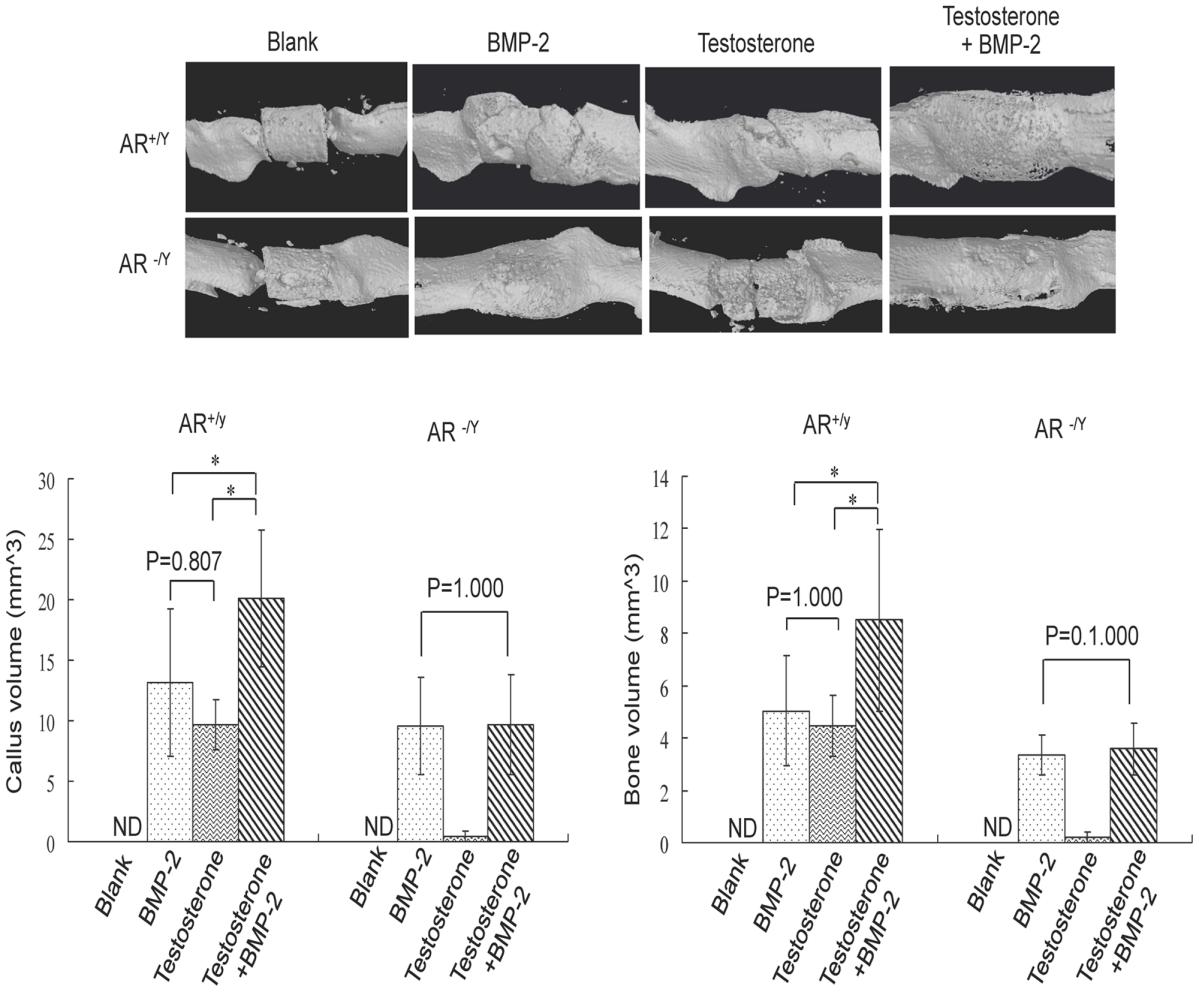
Micro-CT analyses of femurs of wildtype (AR^+/Y^) and ARKO (AR^−/Y^) mice with segmental bone defect treated with a 2.5-mm scaffold containing BMP-2 (5 µg), testosterone (100 µg), or testosterone plus BMP-2 for 35 days. Each femur was scanned for 260 slices each above and below the midpoint of the scaffold for a total of 8.2 mm in length. Upper panel shows reconstructed 3-D images of the region containing the scaffold. The volumes of regenerated callus and bone in the scanned region are shown in column graphs in the lower panel. *: p<0.05. ND: not determined.

Micro-CT analyses were performed at day 35 after surgery. In wildtype mice, no callus or bone formation was detected if the fracture was treated with a scaffold containing no BMP-2 or testosterone. An average of 8 mm^3^, 9 mm^3^, and 16.2 mm^3^ of callus (Tissue Volume, TV) was formed if the scaffold was loaded with BMP-2, testosterone, or testosterone plus BMP-2, respectively (Fig. 3). Similar results in mineralized bone in callus were observed with an average of 4.2 mm^3^, 4 mm^3^ and 6.8 mm^3^ of bone (Bone volume, BV) formed if the scaffold was loaded with BMP-2, testosterone, or testosterone plus BMP-2, respectively. Statistically, there was no difference in effects between BMP-2 and testosterone, whereas treatment with the combination of BMP-2 and testosterone resulted in a significantly higher levels of both callus and BV formation than the treatment with either BMP-2 or testosterone alone (Fig. 3).

In ARKO mice, no callus or bone was formed on the femur treated with blank scaffolds or with those containing only testosterone (Fig. 3). In contrast, an average of 8 mm^3^ and 8.2 mm^3^ of callus was formed if the scaffold was loaded with BMP-2 or testosterone plus BMP-2, respectively (p = 0.772). Similar results were observed with an average of 2.7 mm^3^ and 3.1 mm^3^ bone formed if the scaffold was loaded with BMP-2 or testosterone plus BMP-2, respectively (p = 0.0278). Statistically, there was no difference in effects between BMP-2 and BMP-2 plus testosterone treatments (p>0.05).

Further analyses of the architecture of regenerated calluses showed that the bone volume to callus volume ratios (% BV/TV) in BMP-2, Testosterone (T), and Testosterone+BMP-2 (T+B) groups of wildtype mice were 36.498%, 42.988%, and 39.293%, respectively (Table 2). The average trabecular bone thickness of each of these three groups was 0.084 mm, 0.092 mm, and 0.091 mm, respectively (Table 2). The average trabecular number was 4.290, 4.728, and 4.440 per mm, respectively, and the average trabecular space was 0.159 mm, 0.125 mm, and 0.135 mm, respectively. The structure model indices (SMI), which indicate structural characteristic of bone, of the BMP-2, T, and T+B groups were 2.065, 1.700, and 1.665, respectively. The average cortical BMD at the fracture junctions was 0.370%, 0.381%, and 0.371%, respectively, and the average cortical thickness at the fracture junctions was 0.161, 0.154, and 0.163, respectively. The degree of cortical anisotropy ranged from 2.044 to 2.286. Among these parameters, only the difference in SMI between BMP-2 and T+B groups was statistically significant (p = 0.042). Similar results were obtained with the ARKO mice on the fractures that were treated with scaffolds containing BMP-2 or Testosterone plus BMP-2 (Table 2). Since testosterone had no effect in promoting fracture repair in ARKO mice, these parameters were not measured.

**Table 2:** Micro-CT parameters in femurs of WT (AR^+/Y^) and ARKO (AR^−/Y^) mice with segmental bone defect treated with a 2.5-mm scaffold containing BMP2, testosterone, or BMP2 plus testosterone for 35 days.

	Wildtype			ARKO		
	BMP-2	T	T+B	BMP-2	T	T+B
BV/TV (%)	36.49±7.76	42.998±7.24	39.293±7.84	33.978±8.22	N.D.	31.718±8.11
Tb.Th. (mm)	0.084±0.01	0.092±0.01	0.091±0.01	0.084±0.01	N.D.	0.085±0.01
Tb.N. (mm^−1^)	4.290±1.22	4.728±1.02	4.440±0.89	4.110±0.87	N.D.	4.177±0.83
Tb.Sp. (mm)	0.159±0.09	0.125±0.05	0.135±0.03	0.195±0.05	N.D.	0.189±0.03
SMI	2.065±0.25	1.700±0.35	*1.665±0.20	1.832±0.13	N.D.	1.972±0.23
Cortical BMD (%)	0.370±0.06	0.381±0.06	0.371±0.05	0.317±0.04	N.D.	0.324±0.04
Cortical Th. (mm)	0.161±0.01	0.154±0.02	0.163±0.01	0.136±0.01	N.D	0.135±0.01
Cortical DA	2.044±0.42	2.286 ±0.47	2.057±0.34	2.427±1.23	N.D	2.605±0.46

T: Testosterone; T+B: Testosterone+BMP-2; BV/TV: bone volume/tissue volume; Tb.Th.: trabecular thickness; Tb.N.: trabecular number; Tb.Sp.: trabecular separation; SMI: structure model index; cortical BMD: cortical bone mineral density; cortical Th.: cortical thickness; cortical DA: cortical degree of anisotropy; *: p<0.05; N.D.: not determined.

### Histological examination on effects of BMP-2, testosterone, or combination of both BMP-2 and testosterone on the healing of segmental defect

**Figure 4:**
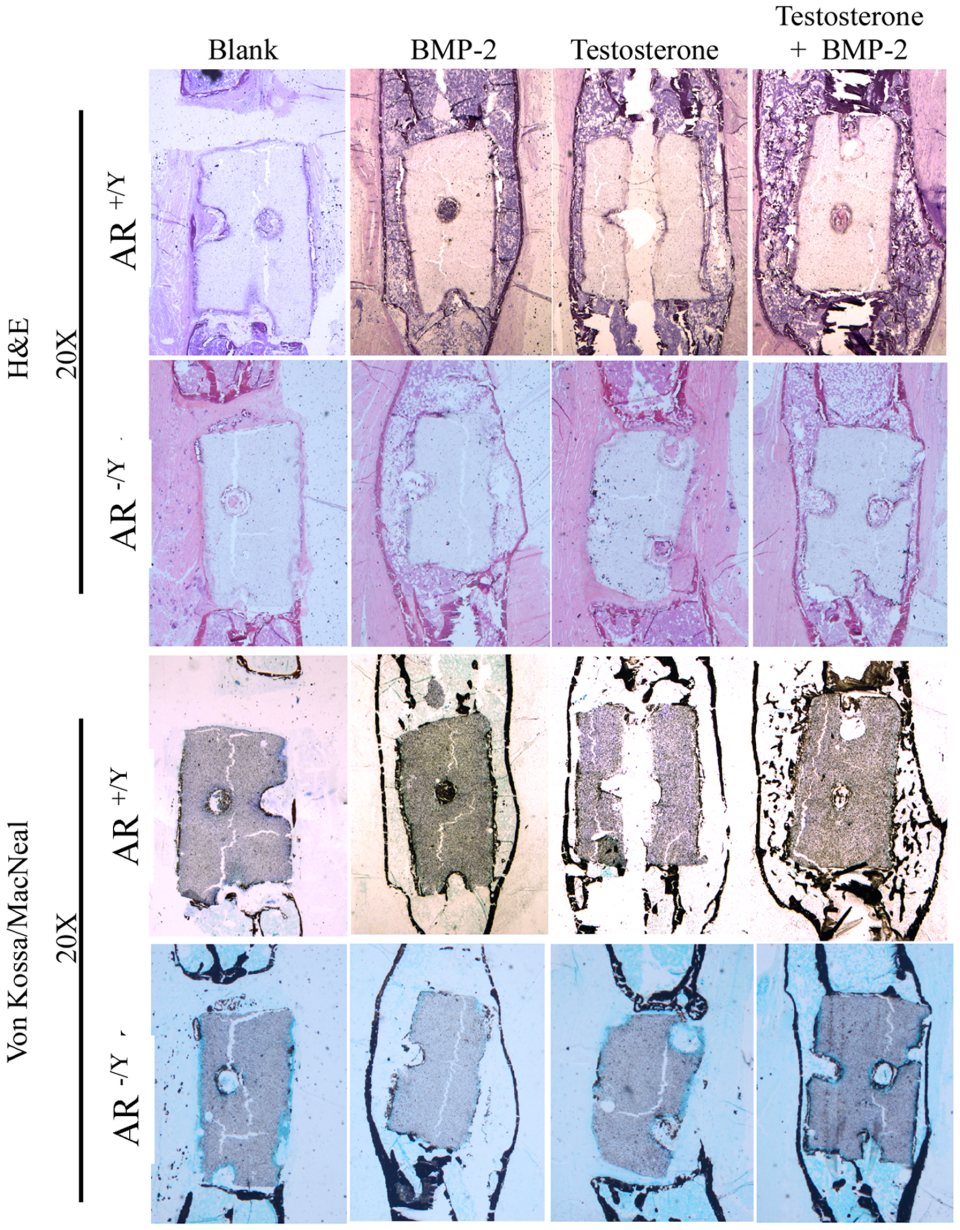
Representative histological images of femurs with segmental defect treated with a 2.5-mm scaffold containing BMP-2 (5 µg), testosterone (100 µg), or testosterone plus BMP-2 at day 35 after osteotomy.

**Figure 5:**
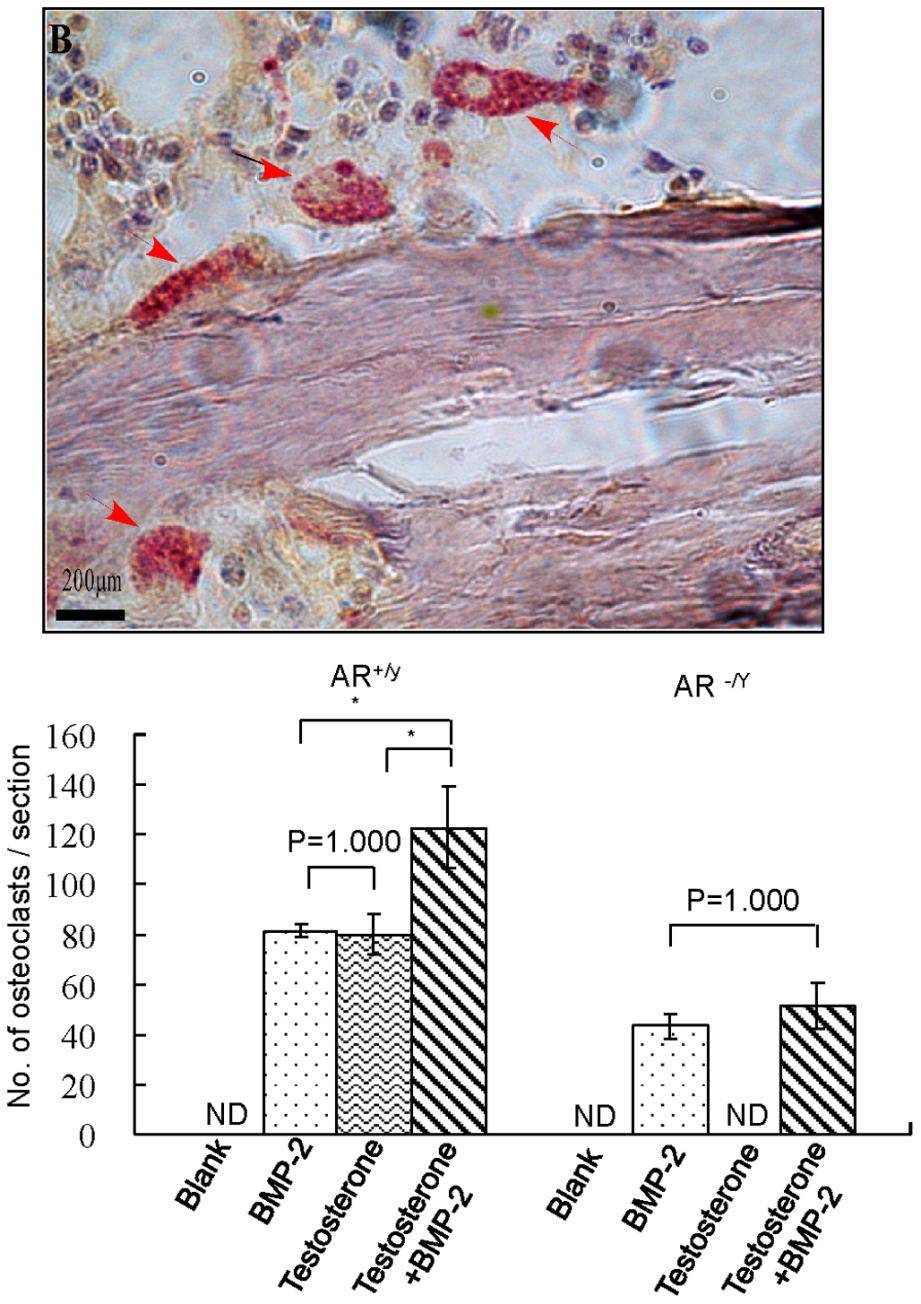
A representative TRAP staining image of femurs with segmental defect treated with a 2.5-mm scaffold containing testosterone, BMP-2 or both at day 35 after osteotomy. Red arrows point to multinucleated osteoclasts. Quantification of osteoclasts (stained in red) in sections from the BMP-2 (5 µg), testosterone (100 µg), or testosterone plus BMP-2 treated groups is shown in the histogram below the image. N.D.: not determined.

Both untreated and treated femurs were examined histologically at day 35 after osteotomy. Longitudinal sections of the region with scaffold were made and stained with H&E or Von Kossa/MacNeal stain. In sections from wildtype mice, both stains showed no bone regeneration in untreated femurs (Fig. 4). H&E staining showed similar degrees of callus formation in BMP-2 or testosterone treated fractures and a much higher degree of callus formation in those treated with both testosterone and BMP-2. Von Kossa/MacNeal staining revealed equal degrees of callus mineralization in BMP-2 and testosterone treated fractures, and a higher degree of callus mineralization in testosterone plus BMP-2 treated femurs. The same results were observed in sections of the femur from ARKO mice treated with scaffolds containing BMP-2 or BMP-2 plus testosterone, but not in those treated with scaffolds containing testosterone only (Fig. 4). TRAP staining showed that approximately 80 osteoclasts were present in each section of the callus of BMP-2 or testosterone treated fracture in wildtype mice (Fig. 5). More (∼120 per section) osteoclasts were detected in the callus of the fracture treated with both BMP-2 and testosterone in wildtype mice. In ARKO mice, approximately equal number (∼40 per section) osteoclasts were detected in the callus of BMP-2 and in BMP-2 plus testosterone treated fracture. Since testosterone had no effect on fracture repair in ARKO mice, TRAP staining was not performed on sections from the fractured femurs of these mice treated with testosterone only.

### Systemic effects of locally applied BMP-2 or testosterone

To determine whether local administration of BMP-2 or testosterone had any systemic effect, the weights of body, seminal vesicles, testis, and prostate at day 35 after treatment were measured. In wildtype mice, the body weights of testosterone-treated mice were lower (24.338 g vs. 25.490 g) than those of untreated mice (Table 3). There was no profound difference in body weight between BMP-2 and testosterone plus BMP-2 treated mice (25.266 g vs. 25.672 g). However, BMP-2 treated mice were slightly heavier than those treated with testosterone (25.266 g vs. 24.338 g). Weights of seminal vesicles were 0.148 g, 0.155 g, 0.162 g, and 0.149 g for Blank, BMP-2, Testosterone, and BMP-2+Testosterone groups, respectively. The average testis weight of the BMP-2 treated mice was the highest (0.127 g), followed by that of testosterone treated (0.118 g) and then those of testosterone plus BMP-2 treated (0.113 g) and untreated mice (0.093 g). There was no significant difference in prostate weight among all groups (range: 0.046 g–0.059 g). Although there were variations in these parameters, none of the differences were statistically significant. Similar results were obtained from ARKO mice. The body weights ranged from 21.154 g to 22.146 g, and those of testes ranged from 0.012 g to 0.019 g with no significant difference between the 4 groups.

**Table 3:** Average weights of body, testis, seminal vesicles, and prostate of mice in each group at day 35 after various treatments.

	Wildtype				ARKO			
	Blank	B	T	T+B	Blank	B	T	T+B
Body (g)	25.490±2.158	25.266±3.249	24.338±2.399	25.672±1.323	21.306±1.403	22.146±2.145	21.644±0.715	21.154±1.050
Seminal vesicles (g)	0.148±0.026	0.155±0.032	0.162±0.025	0.149±0.030	N.D.	N.D.	N.D.	N.D.
Testis (g)	0.093±0.043	0.127±0.045	0.118±0.054	0.113±0.042	0.019±0.005	0.013±0.004	0.016±0.004	0.012±0.002
Prostate (g)	0.048±0.007	0.046±0.010	0.049±0.009	0.059±0.018	N.D.	N.D.	N.D.	N.D.

B: BMP-2; T: Testosterone; T+B: Testosterone+BMP-2; N.D.: not determined.

Examination of the area under the growth plate of right femur (un-fractured control) of each mouse showed no significant differences in the ratio of bone and tissue volumes; trabecular bone thickness, trabecular number, space, and pattern factor; structure model index (SMI), cortical BMD, and cortical thickness (Table 4). In wildtype mice, the ratios of bone and tissue volumes (BV/TV) in the Blank, BMP-2, T, and T+B groups were 33.311%, 38.644%, 29.967%, and 30.068%, respectively. The average trabecular bone thickness (Tb. Th.) of each of these four groups was 0.083 mm, 0.081 mm, 0.078 mm, and 0.076 mm, respectively. The average trabecular number (Tb. N.) of each of these groups was 4.037, 4.623, 3.653, and 4.034 per mm, respectively. The average trabecular space of the groups was in the range between 0.157 mm and 0.195 mm. The SMI for each of these 4 groups was 4.634, 5.867, 4.310, and 3.562, respectively. Average cortical BMD of each group was 0.619%, 0.632%, 0.607%, and 0,607%, respectively. The average cortical thickness was in the range of 0.201–0.215, and the degree of cortical anisotropy ranged from 2.200 to 2.600. Very similar results were obtained with the ARKO mice. None of the values of the various treatment groups were statistically significant when compared to that of the control (Blank) group.

**Table 4:** Effects of BMP-2 or testosterone on control un-fractured femur.

	Wildtype				ARKO			
	Blank	BMP-2	T	T+B	Blank	BMP-2	T	T+B
BV/TV (%)	33.31±5.17	38.644±5.61	29.967±3.68	30.068±6.16	18.062±1.89	18.279±2.43	17.400±3.17	18.804±2.18
Tb.Th. (mm)	0.08±0.01	0.081±0.01	0.078±0.01	0.076±0.01	0.057±0.01	0.064±0.01	0.064±0.01	0.066±0.01
Tb.N. (mm^−1^)	4.03±0.59	4.623±0.64	3.653±0.32	4.034±0.71	2.969±0.38	2.824±0.20	2.589±0.35	2.890±0.41
Tb.Sp. (mm)	0.18±0.02	0.157±0.03	0.182±0.02	0.195±0.06	0.247±0.09	0.322±0.08	0.334±0.04	0.342±0.11
SMI	4.63±0.36	5.867±2.95	4.310±1.45	4.310±1.45	8.632±1.04	6.832± 0.85	5.797±3.14	6.925±2.67
Cortical BMD (%)	0.61±0.09	0.632±0.06	0.607±0.09	0.607±0.08	0.594±0.06	0.594±0.12	0.632±0.07	0.606±0.11
Cortical Th. (mm)	0.21±0.02	0.207±0.02	0.201±0.02	0.206±0.01	0.183±0.02	0.189±0.01	0.197±0.01	0.190±0.01
Cortical DA	2.22±0.30	2.299±0.15	2.600±0.28	2.200±0.24	2.420±0.23	2.328±0.25	2.456±0.18	2.532±0.25

T: Testosterone; T+B: Testosterone+BMP-2; BV/TV: bone volume/tissue volume; Tb.Th.: trabecular thickness; Tb.N.: trabecular number; Tb.Sp.: trabecular separation; SMI: structure model index; cortical BMD: cortical bone mineral density; cortical Th.: cortical thickness; cortical DA: cortical degree of anisotropy.

## Discussion

Fracture repair involves all the processes in bone development including proliferation, differentiation, and mineralization of osteoblasts. Critical-size defect fractures do not heal completely without the aid of mechanical supports and growth factors. The use of BMP-2 loaded scaffold to stabilize the fracture and to initiate bone regeneration has been shown to be an effective treatment for segmental defects [6]. Although BMP-2 has high osteoinductive potency and can improve bone healing, its high cost and potential adverse effects make this approach unfavorable. Since testosterone has anabolic effects on bone development, we tested its effects on the repair of critical-size segmental fractures and compared its efficacy to that of BMP-2. We found that treating the fracture with a scaffold containing BMP-2 resulted in callus formation 14 days after initiation of the treatment, whereas no callus was formed if the fracture was treated with a scaffold containing no BMP-2 or testosterone during the entire period (35 days) of the study (Fig. 1, 2). Interestingly, similar results were observed at the same time (day 14) if the fracture was treated with a scaffold containing testosterone (Fig. 1). Results of micro-CT examinations showed that the degrees of callus formation and bone regeneration were comparable between testosterone and BMP-2 treated fractures (Fig. 3). Analyses of structure model index (SMI) revealed that the fractures treated with testosterone plus BMP-2 (1.665) had a significant lower SMI (SMI = 1.665) than that treated with BMP-2 alone (2.065) (Table 2), suggesting that testosterone plus BMP2 is more efficacious than BMP-2 alone in promoting bone regeneration. Histological examinations on both trabecular and cortical bone showed no significant difference between BMP-2 and testosterone treated fractures (Fig. 4). Together, these results suggest that testosterone is as effective as BMP-2 in promoting the healing of critical-size segmental defects of femoral bone in mice.

BMP-2 is known to activate bone regeneration by two different mechanisms, depending on the type of receptors bound. Two different BMP-2 receptor proteins, types I and II, with serine/threonine kinase activity exist. A functional BMP-2 receptor is a heterodimer of these two receptor proteins. Type II receptor phosphorylates type I receptor which then activates Smad 1, 5, and 8 [35]–[38]. These activated Smad proteins interact with Runx-2/Cbfa-1 to promote maturation of chondrocytes [39]. Binding of BMP-2 to its receptor also induces formation of more heterodimeric receptors. When BMP-2 binds to these newly formed receptors, it activates the MAPK pathway [40], leading to activation of many transcription factors such as members of the Fos/Jun family and the activating transcription factor-2 and subsequently increased production of fibronectin and osteopontin [41]. Androgens have been shown to modulate the expression of genes in the BMP signaling pathway including genes for chordin, SMAD specific E3 ubiquitin protein ligase 1 (Smurf1), Six3, and sclerostin [42]. In this study, we found that BMP-2 promoted bone healing in ARKO mice, suggesting that BMP-2 can be used to aid fracture repairs in individuals deficient in the androgen/AR pathway. It is likely that BMP-2 and testosterone promote bone regeneration by different mechanisms. Since we found that combination therapy with testosterone and BMP-2 was superior to single therapy (Figs. 1–3), these mechanisms may be complementary or synergistic leading to a more speedy fracture healing.

Testosterone may affect bone [43] development directly or indirectly by being converted to estrogen through aromatization. In direct action, testosterone binds to and activates AR, enabling it to be transported into the nucleus to bind to the androgen response element. As a result, the transcription of many target genes such as the osteoblast genes *AKP2*, *Colla1*, and *Bglap* are activated to promote bone mineralization [21], [44]. Testosterone also has nongenomic effects by activating PI3K/Akt signaling pathways in osteoblasts [20], [45] and altering the activity of Elk-1, CCAAT enhancer binding protein-β, and cAMP-response element binding protein resulting in activation of the Src/Shc/ERK pathway. Testosterone can also interact with c-Jun/c-Fos leading to down-regulation of the c-Jun N-terminal kinase. Since these actions are anti-apoptotic, they are beneficial to the differentiation of osteoblasts. Testosterone may also affect bone regeneration by increasing the production of TGF-β and IGFs and decreasing the production of IL-6 receptors [46]–[49]. IGFs and IGF-binding proteins can enhance osteoblast proliferation and differentiation [50], [51]. In addition to the anabolic effect, testosterone is also anti-resorptive as a decline in bone resorption is seen in hypogonadal men after testosterone replacement [52]–[56]. It is possible that these pathways all play important roles at different stages of bone healing. However, it remains unclear as to how androgens and AR coordinate the expression of genes in these pathways. We have also detected the presence of osteoclasts in regenerating calluses by TRAP staining (Fig. 5). Since bone remodeling is the balance in action between osteoblasts and osteoclasts [57], it is conceivable that the osteoclasts were present in the regenerating calluses. We also found that the number of osteoclasts in the calluses of fractures treated with both testosterone and BMP-2 were significantly higher (∼120 vs. 80 per section) than those treated with either alone (Fig. 5). This result suggests that osteoclastic activities were promoted by both testosterone and BMP-2 during bone fracture healing.

Testosterone has different effects on different types of skeletal cells. It has been shown to decrease osteoblast and osteocyte apoptosis [58], [59], stimulate proliferation of osteoblast progenitors and differentiation of mature osteoblasts, and promote the apoptosis of osteoclasts [58], [59] and epiphyseal growth and maturation [60]. Using AR transgenic mice under the control of the 2.3-kb alpha (I)-collagen promoter, Wiren et al. showed that anabolic effects of testosterone exhibited exclusively on periosteal surfaces, suggesting that the consequences of androgen action could be compartment specific [61]. While AR over expression has been postulated to limit the effect of androgen on osteoblast differentiation and mineralization [42], our results suggest that local short-term testosterone treatment can initiate bone regeneration.

Since no callus formation on the fractured femur treated with a scaffold containing testosterone in ARKO mice was observed during the entire 35-day period of the study (Fig. 2), it is likely that testosterone promotes bone regeneration through the genomic instead of the nongenomic pathway by activating AR. This is the first finding that AR is required for testosterone to promote bone fracture repair. This result also suggests that estrogen derived from aromatization of testosterone plays little role in fracture repair. However, this possibility requires to be verified. Since this study was done in male mice, the effects of testosterone and the roles of AR in promoting fracture healing in females remain to be investigated.

The results of this study strongly suggest that testosterone can be used to promote fracture healing. This finding is consistent with that described by Zaifirau et al. [62] who found that traumatized bone of Sprague-Dawley rats implanted with twelve-hour calcined hydroxyapatide ceramics containing testosterone healed faster than the controls. Similarly, Gordon et al. [63] reported that a self-setting zinc sulfate calcium phosphate containing testosterone was integrated into traumatized femurs of albino Holtzman rats. Benghuzzi et al. [64] found that delivery of dihydrotestosterone using the tricalcium phosphate lysine delivery system to fractured femurs of Sprague-Dawley rats resulted in stimulation of osteoblastic activities and increased cortical bone density. The tricalcium phosphate lysine system was also used to deliver simvastatin (inhibitor of the 3-hydroxy-3-methylglutaryl coenzyme A reductase) to promote the healing of segmental bone fractures [65]. Although all of these studies found testosterone to be effective in promoting fracture repair, its efficacy was not clear because it was not compared to that of any well-established treatment method. In this study, we compared the efficacy of testosterone for fracture repair with that of BMP-2 which is FDA approved for treatment of acute, open tibial shaft fractures as described above and found that testosterone is as effective as BMP-2 in promoting fracture healing. We also used scaffolds that have been shown to effectively deliver BMP-2 and antibiotics to fractured bone [6], [66]. Although the scaffolds used to bridge the fractures in this study were not resorbed during the 35-day period of the investigation, their biodegradable nature had been firmly established [67], [68] and had been shown to be resorbed in 6 months in a canine model [69]. This slow resorption rate of the scaffold is beneficial as it provides sufficient time for the fractured bone to heal to the point where it can stand weights.

Although testosterone was found to be effective in promoting fracture repair, it has not been used clinically to treat fractures, perhaps due to the concern over its virilizing side effects as well as the possibility of altering lipoprotein profiles and increasing the levels of endothelin-1, C-reactive protein, and total homocysteine [70]. However, we found that the dose of testosterone required to promote fracture repair is very low. In this study, we used 100 µg and loaded it onto a scaffold used to bridge the fracture on femur in mice. Since mice are 12 times more resistant to drugs than humans [30], only 8.3 µg of testosterone would be required to treat a segmental defect of the same size in humans and only approximately 1.8 ng/ml increase in plasma testosterone levels would result if all of it is absorbed at once. This would not significantly alter the physiological levels of testosterone. Therefore, adverse effects would be minimal as evidenced by our demonstration of the lack of effects on BMD, seminal vesicles, testis, and prostate (Table 4). We also found that the combination of BMP-2 and testosterone works better than either BMP-2 or testosterone alone. Since testosterone is much cheaper than BMP-2, its short-term local application in fracture treatment would be more feasible. As increasing number of adverse effects are found to be associated with BMP-2 [13], alternative treatments are needed. Our findings may provide a more economic method using testosterone or a more efficient means using a combination of BMP-2 and testosterone to treat fractures with critical-size segmental defects of a long bone.
